# Baseline cell cycle and immune profiles indicate CDK4/6 inhibitor response in metastatic HR + /HER2- breast cancer

**DOI:** 10.1038/s41523-025-00767-2

**Published:** 2025-06-12

**Authors:** Stephanie L. Tzetzo, Emily Schultz, Jianxin Wang, Hanna R. Rosenheck, Sidney Mahan, Erik S. Knudsen, Agnieszka K. Witkiewicz

**Affiliations:** 1 Department of Molecular and Cellular Biology, Roswell Park Comprehensive Cancer Center, Buffalo, NY USA; 2 Department of Pathology, Roswell Park Comprehensive Cancer Center, Buffalo, NY USA

**Keywords:** Cancer microenvironment, Cancer therapy

## Abstract

While CDK4/6 inhibitors (CDK4/6i) and endocrine therapy are standard-of-care for metastatic HR + /HER2- breast cancer, patient selection for durable efficacy remains undefined. Here, we assessed baseline cell cycle and immune profiles in a CDK4/6i-treated patient cohort with differential progression-free survival (PFS < 6 months vs. >23 months) using transcriptomic and protein-based imaging approaches. Cell cycle, polo-like kinase signaling and transcription gene sets are largely enriched among pre-treatment tissue of patients with short PFS. Pre-treatment tumors express cyclin A or E significantly higher in patients with short PFS and correlate with macrophage accumulation. Patients with long PFS display gene set enrichment for growth factor and immune signaling pre-treatment, while gene set enrichment for immune activation emerges during CDK4/6i therapy. Our data highlight baseline tumor-intrinsic and tumor microenvironments-associated indicators of CDK4/6i response in the “real-world” setting and offer implications for precision-based therapeutic combinations to enhance CDK4/6i efficacy. Clinical trial registration number: NCT04526587.

## Introduction

The most frequently diagnosed breast cancer subtype is hormone receptor-positive and HER2-negative (HR + /HER2-)^1^. While early-stage HR + /HER2- breast cancer commonly associates with favorable prognosis, recurrent or de novo metastatic disease remains the main cause of cancer-related death^2^. As HR + /HER2- disease is highly dependent on estrogen for tumor growth, standard-of-care treatment relies on endocrine therapy involving inhibition of estrogen-mediated signaling through the usage of tamoxifen, aromatase inhibitors (AI) or selective estrogen degraders (SERD) such as fulvestrant^3^. Endocrine therapy may delay progression of metastatic disease, but most patients become resistant to first-line endocrine therapy. Combining endocrine therapy with cyclin-dependent kinase 4/6 inhibitors (CDK4/6i) including palbociclib, ribociclib or abemaciclib significantly increases progression-free survival (PFS) and are FDA-approved agents for metastatic HR + /HER2- breast cancer^1,4,5^. However, this therapeutic combination remains not curative for metastatic disease^6^.

Tumor-intrinsic mechanisms contributing to CDK4/6i therapeutic resistance often include cell cycle dysregulation^7,8^. RB is instrumental for CDK4/6i efficacy as a tumor suppressor and inhibitor of cell cycle entry. Clinical loss of RB has been observed during progression on CDK4/6i-based therapy^9,10^. Other cell cycle-associated resistance mechanisms may arise without altering RB expression such as amplification of CDK6 or cyclin E^9–12^. Additionally, upregulation of growth factor receptors such as HER2, FGFR2 or EGFR and intracellular signaling involving mTOR may further stimulate cell cycle entry^8,10,13^.

Alongside tumor-intrinsic resistance mechanisms, immune cells within the tumor microenvironment (TME) may influence CDK4/6i efficacy^14^. Preclinical and clinical studies have indicated a potent role for CDK4/6i therapy in promoting antitumor immune responses through activation of CD8^+^ T cells and reduction of immunosuppressive regulatory T cells (Tregs)^15–17^. A recent clinical assessment suggested that overall low baseline or reduced blood lymphocyte levels during CDK4/6i treatment predict shorter PFS^18^. Another clinical study suggested that response to CDK4/6i associated with a reduction in circulating Tregs and an increase in CD8^+^ T cells^19^. While such peripheral immune assessments suggest the importance of an endogenous immune response during therapy, cell cycle and immune biomarkers within the TME that indicate patient selection for long PFS with CDK4/6i therapy remain to be clinically evaluated.

Here, we demonstrate tumor cell-intrinsic and stromal indicators of durable CDK4/6i response through transcriptomic and multispectral immunofluorescent (mIF) imaging analyses of distinct patient cohorts with short (<6 months) or long (>23 months) PFS in response to CDK4/6i at a single cancer center. Gene enrichment for cell cycle, polo-like kinase signaling and transcription was upregulated in pre-treatment tissue of patients with short PFS while multiple growth factor- or immune-mediated signaling gene sets were downregulated in comparison to patients with long PFS. Our imaging analyses revealed that low protein levels of cyclin A or E among pre-treatment tumors associate with long PFS on CDK4/6i-based therapy. We demonstrate a “real-world” association of tumor-intrinsic cyclin levels on the surrounding TME as pre-CDK4/6i tumor expression of cyclin E strongly correlates with macrophage abundance and on-treatment cyclin E^+^ tumors inversely correlate with CD8^+^ T cell detection. Furthermore, transcriptomic and imaging analyses were performed on two independent cohorts of on-treatment biopsies which collectively indicated cell cycle reduction and an emergence of gene enrichment for T cell activation during CDK4/6i-based therapy.

## Results

### Identification of a patient cohort with exceptional treatment response

**Fig. 1:**
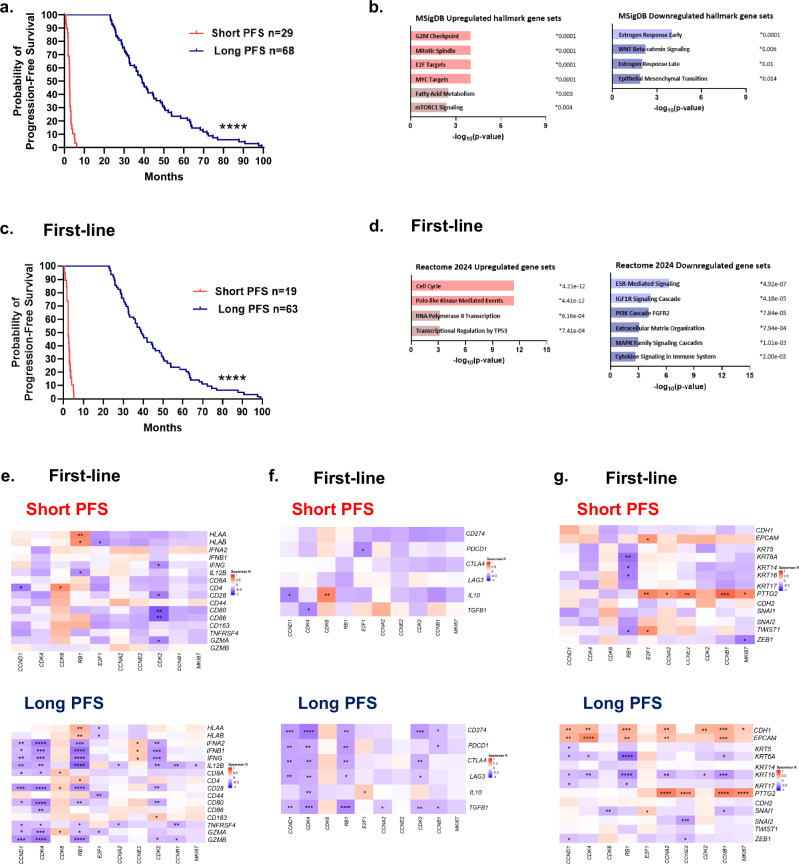
Evaluation of an exceptional patient response cohort to CDK4/6i-based therapy. **a** Assessment of progression-free survival (PFS) of patients treated with CDK4/6 inhibitors (CDK4/6i) and classified as short (<6 months; *n* = 29) or long (>23 months; *n* = 68) PFS. **b** GSEA of pretreatment tissue from patients with short PFS compared to patients with long PFS. **c** PFS of patients treated with CDK4/6i first-line. **d** Enrichr plots of pre-treatment gene sets upregulated (left) or downregulated (right) in first-line patients with short PFS compared to patients with long PFS. **e–g** Correlations of cell cycle transcripts with immune-stimulatory (**e**), immunosuppressive (**f**), or epithelial and mesenchymal (**g**) gene expression in pre-treatment tissue of patients with short (top) or long (bottom) PFS. Significance was determined by log rank test (**a**, **c**), Fisher’s exact test (**b**, **d**) or Spearman correlation (**e**–**g**; *n* ≥ 17 patients/group). *p < 0.05; **p < 0.01; ***p < 0.001; ****p < 0.0001.

A clinical study at Roswell Park Comprehensive Cancer Center was developed to assess features of HR + /HER2- breast cancer treated with CDK4/6i as standard-of-care (NCT04526587)^5^. All patients provided written informed consent for this IRB-approved study (protocol #I-571719). Clinical and pathological features were collected from patient chart review and documented within an electronic medical database (REDCap). 155 patients with pre-treatment tissue from our trial were reviewed to stratify PFS on CDK4/6i-based therapy to dichotomize patient response (Supplementary Table 1). We defined long PFS as >23 months based on evidence from multiple clinical trials that demonstrate an approximate 2-year median PFS of a CDK4/6i combined with an aromatase inhibitor and slightly less than 2-year median PFS of a CDK4/6i in combination with fulvestrant^5,20–24^. Short PFS was defined as <6 months in part due to the demonstrated efficacy of approximately 9.5 months median PFS of palbociclib with fulvestrant in the PALOMA-3 trial^25^. Our selected dichotomy offers a rigorous approach to define exceptional treatment response unlike current “real-world” assessments of long-term response that are based on broad patient cohort responders^26,27^. We identified 29 patients with short PFS that ultimately progressed on therapy (100%) and 68 patients with long PFS that remain (47.1%) or have progressed (45.6%) on therapy (Table 1). The two arms had a median PFS of 2.60 and 39.12 months and were significantly different as expected (Fig. 1a). Short and long PFS cohorts were mainly treated with palbociclib and in first-line settings (Table 1). Endocrine therapy administered as standard-of-care with CDK4/6i was mainly composed of aromatase inhibitors (letrozole, anastrozole or exemestane) in patients with long PFS (91.2%). Patients with short PFS were treated with aromatase inhibitors (48.3%) or fulvestrant (51.7%). Visceral metastatic disease was prevalent in pre-CDK4/6i treatment settings in patients with short PFS while patients with long PFS had similar percentages of non-visceral or visceral metastasis (Table 1). More than half of patients with short PFS had prior treatment, consistent with our previous report that prior endocrine therapy or chemotherapy significantly reduce PFS on CDK4/6i-based therapy^11^. An unbiased assessment of the stratified PFS cohorts demonstrated pre-treatment gene set enrichment for cell cycle, MYC, fatty acid metabolism, or mTORC1 signaling pathways in patients with short PFS while estrogen, WNT beta-catenin or epithelial-mesenchymal transition (EMT) signaling were enriched in patients with long PFS (Fig. 1b).

**Table 1:** Characteristics of patients stratified according to PFS on CDK4/6i-based therapy

Patient Characteristics	Short PFS (*n* = 29)	Long PFS (*n* = 68)
Months PFS (median)	2.60	39.12
CDK4/6i therapy (%)		
Palbociclib	27 (93.1)	65 (95.6)
Abemaciclib	0 (0.0)	1 (1.5)
Ribociclib	2 (6.9)	2 (2.9)
First-line	19 (65.5)	63 (92.6)
CDK4/6i status (%)		
Still on	0 (0.0)	32 (47.1)
Off due to progression	29 (100.0)	31 (45.6)
Off due to adverse effects	0 (0.0)	4 (5.8)
Off due to death	0 (0.0)	1 (1.5)
Endocrine therapy (%)		
Aromatase inhibitor	14 (48.3)	62 (91.2)
Fulvestrant	15 (51.7)	5 (7.3)
Tamoxifen	0 (0.0)	1 (1.5)
Race (%)		
African American	3 (10.3)	5 (7.3)
Caucasian American	23 (79.3)	62 (91.2)
Other	2 (6.9)	0 (0.0)
Not reported	1 (3.5)	1 (1.5)
Age at CDK4/6i start (mean)	61.59	65.24
Menopause status (%)		
Premenopausal	6 (20.7)	8 (11.8)
Menopausal	23 (79.3)	60 (88.2)
Histologic diagnosis (%)		
Invasive ductal carcinoma	22 (75.9)	50 (73.5)
Invasive lobular carcinoma	5 (17.2)	13 (19.1)
Invasive mixed carcinoma	2 (6.9)	3 (4.4)
Invasive carcinoma with micropapillary features	0 (0.0)	1 (1.5)
Invasive solid papillary carcinoma	0 (0.0)	1 (1.5)
Metastatic status at presentation (%)		
De novo	7 (24.1)	28 (41.2)
Recurrent	22 (75.9)	40 (58.8)
Metastatic status (%)		
Non-visceral	6 (20.7)	40 (58.8)
Visceral	23 (79.3)	28 (41.2)
Prior chemotherapy (%)	19 (65.5)	29 (42.6)
Prior endocrine therapy (%)	23 (79.3)	33 (48.5)
Prior radiation therapy (%)	23 (79.3)	28 (41.2)
Surgical excision (%)	13 (44.8)	25 (36.8)
Mastectomy (%)	11 (37.9)	16 (23.5)

Demographic, treatment and disease status of metastatic HR + /HER2- breast cancer patients with short (<6 months) or long (>23 months) PFS on CDK4/6i therapy. *CDK4/6i* CDK4/6 inhibitor; *PFS* progression-free survival.

To identify differential pre-treatment gene expression of the exceptional response cohort, we focused targeted RNA-seq analyses on patient cohorts treated with CDK4/6i first-line to reduce confounding variables from additional treatment (Fig. 1c and Supplementary Table 2). Based on the Reactome 2024 pathway database, gene sets associated with cell cycle, polo-like kinase activity and transcription were significantly upregulated in pre-treatment tumor biopsies of patients with short PFS (Fig. 1d) along with *CCNB1* and *MKI67* transcripts (Table 2). Gene sets associated with growth factor-mediated signaling (ESR, IGF1R, PI3K, FGFR2, MAPK) or extracellular matrix (ECM) organization were significantly downregulated in pre-treatment tissue of patients with short PFS (Fig. 1d). *FGFR2* and *IGFR1* transcripts were significantly higher in pre-CDK4/6i tumor biopsies of patients with long PFS. High *IGF1R* gene expression associated with improved PFS among the combined first-line cohort (Table 2). Furthermore, pre-treatment gene enrichment for immune processes involving cytokine signaling were also downregulated in patients with short PFS (Fig. 1d).

**Table 2:** Pre-treatment cell cycle, immune, growth factor receptor or epithelial gene expression, distribution and associations with PFS

		Total PFS cohort				First-line PFS cohort			
		Mean gene expression		Gene association with PFS of the combined cohort (*n* = 95)		Mean gene expression		Gene association with PFS of the combined cohort (*n* = 80)	
Pre-treatment gene set	Pre-treatment gene	Short PFS (*n* = 27)	Long PFS (*n* = 68)	HR	*p*-value	Short PFS (*n* = 17)	Long PFS (*n* = 63)	HR	*p*-value
Cell cycle	*CCND1*	11.2	11.08	1.02	0.8	11.27	11.08	1.00	1.00
	*CDK4*	8.9	8.66	1.76	0.08	8.79	8.67	1.42	0.3
	*CDK6*	7.06	6.87	1.43	0.06	6.88	6.86	1.31	0.2
	*RB1*	8.02	8.00	1.03	0.8	8.08	8.01	1.05	0.8
	*E2F1*	6.62	6.28	**1.57**	*0.01	6.50	6.30	1.44	0.08
	*CCNA2*	7.62**	7.11	**1.6**	*0.03	7.52	7.14	1.31	0.3
	*CCNE2*	7.71*	7.33	**1.56**	*0.03	7.61	7.35	1.30	0.3
	*CDK2*	8.06	8.01	1.03	0.9	7.97	8.03	0.68	0.4
	*CCNB1*	8.56**	7.97	**1.56**	*0.03	8.43*	8.00	1.21	0.4
	*MKI67*	7.82**	7.16	**1.6**	*0.01	7.68*	7.18	1.37	0.1
Immune	*CD163*	9.22	8.64	**1.32**	*0.02	9.25	8.61	1.29	0.05
	*CD3d*	6.23	5.97	0.9	0.4	6.04	6.18	0.98	0.9
	*CD8a*	6.57	6.59	0.96	0.7	6.71	6.58	1.03	0.8
	*FOXP3*	3.83	4.14	1.01	1.0	3.85	4.09	1.06	0.6
	*TNFRSF4*	4.5	4.93*	0.89	0.3	4.47	4.90	0.92	0.5
	*PDCD1*	4.36	4.45	1.02	0.9	4.45	4.42	1.12	0.4
	*CD274*	5.82	5.71	1.29	0.1	5.81	5.68	1.36	0.1
Growth factor receptor	*ESR1*	10.86	11.48	0.93	0.3	10.57	11.44	0.87	0.1
	*ESR2*	4.16	4.28	1.10	0.4	4.17	4.17	1.17	0.2
	*FGFR2*	7.22	7.85*	0.86	0.3	7.15	7.79*	0.79	0.1
	*FGFR4*	6.69**	5.83	1.15	0.09	6.4	5.88	1.07	0.5
	*IGFR1*	8.14	9.35***	**0.7**	***0.0003	8.13	9.36**	**0.71**	**0.002
	*PI3KR*	9.26	9.73*	0.77	0.05	9.3	9.76	0.79	0.1
	*PGR*	5.55	6.82*	**0.87**	*0.02	6.24	6.76	0.93	0.3
Epithelial	*EPCAM*	9.14*	8.7	1.12	0.4	9.03	8.72	1.02	0.9
	*KRT14*	4.55	5.66*	**0.86**	*0.03	5.24	5.65	0.89	0.1
	*PTTG2*	7.66**	7.08	**2.14**	***0.0008	7.73**	7.09	**2.05**	**0.005
	*ZEB1*	7.31	7.64*	0.63	0.06	7.34	7.65*	0.70	0.2

Mean gene expression was determined by targeted RNA-seq analyses to calculate differential gene expression and association with PFS. Differential gene expression of the total stratified PFS cohort (left) or the first-line CDK4/6i (right) patient cohort with short or long PFS was determined by Wald tests and significant differential gene expression is bolded. Univariate Cox proportional-HR determined pre-treatment gene associations with PFS of the combined short and long patient cohorts. Significant gene associations are bolded. *p < 0.05; **p < 0.01; ***p < 0.001. *HR* hazard regression, *PFS* progression-free survival.

As our prior work recognized the impact of various cell cycle gene signatures on reduced CDK4/6i-based efficacy^11^, we further examined cell cycle transcripts in relation to immune or epithelial gene expression. Pre-treatment cell cycle transcripts displayed similar correlations in first-line patients with short or long PFS in which *MKI67* strongly correlated with *CCNA2*, *CCNE2*, *CDK2*, or *CCNB1* (Supplementary Fig. 1a). An immune-stimulatory gene panel was selected to assess various genes involved in antitumor activity characteristic of CD8^+^ T cells including MHC class I molecules, interferons, costimulatory molecules, and granzyme^28,29^ (Supplementary Fig. 1b). Pre-treatment expression of *CDK2* inversely correlated with *IFNG*, costimulatory molecule (*CD28*, *CD80*) and granzyme genes while *RB1* correlated with MHC class I molecules (*HLA-A/B*) in patients with short or long PFS (Fig. 1e). Interestingly, *CCNE2* correlated with type I (*IFNA2*, *IFNB1*) and type II (*IFNG*) interferons while *CDK2* correlated with the pan-macrophage *CD163* transcript in patients with long PFS (Fig. 1e). We then compared immune checkpoint molecule (*CD274*, *PDCD1*, *CTLA4*, *LAG3*) and immunosuppressive cytokine (*IL10*, *TGFB1*)^28^ gene expression (Supplementary Fig. 1c). Immunosuppressive cytokines inversely correlated with *CCND1* or *CDK4* in patients with short or long PFS (Fig. 1f). In patients with short PFS, *CDK6* correlated with *IL10* while *E2F1* negatively associated with *PDCD1*. *E2F1* associated with *IL10* among patients with long PFS while *CCND1*, *CDK4*, *RB1*, *CDK2*, or *CCNB1* negatively correlated with multiple immune checkpoint molecule transcripts. Overall, pre-CDK4/6i cell cycle transcripts mostly displayed negative correlations with immune-stimulatory or immunosuppressive genes.

We then focused on correlations of pre-treatment cell cycle transcripts with epithelial (*CDH1*, *EPCAM*, *PTTG2*), cytokeratin or mesenchymal (*CDH2*, *SNAI1/2*, *TWIST1*, *ZEB1*) gene expression to assess EMT^30,31^. *PTTG2* was significantly higher in patients with short PFS while patients with long PFS had greater expression of *ZEB1* (Table 2). *ZEB1* directly correlated with *SNAI2* and *TWIST1* in first-line patients (Supplementary Fig. 1d). In patients with short PFS, *E2F1*, *CCNA2*, *CCNE2*, *CCNB1* and *MKI67* correlated with *PTTG2* while *RB1* negatively correlated with *TWIST1* and multiple cytokeratin transcripts (Fig. 1g). Patients with long PFS displayed strong correlations of *EPCAM*, *CDH1* and *PTTG2* with *CCNA2* and *CCNB1*. *KRT6A* and *KRT16* cytokeratin transcripts inversely correlated with *CCND1*, *CDK4*, *RB1*, and *CCNB1* transcripts in patients with long PFS. Overall, pre-CDK4/6i cell cycle transcripts mostly correlated with *EPCAM* or *PTTG2* while inversely correlating with cytokeratin or mesenchymal genes. High *PTTG2* gene expression also associated with worse PFS among the combined first-line cohort (Table 2). Finally, pre-treatment correlations of the total stratified PFS cohort displayed similar gene expression as the first-line cohort (Table 2 and Supplementary Fig. 1e–h).

### Interrogation of cell cycle and immune cell profiles within pre-treatment patient biopsies

**Fig. 2:**
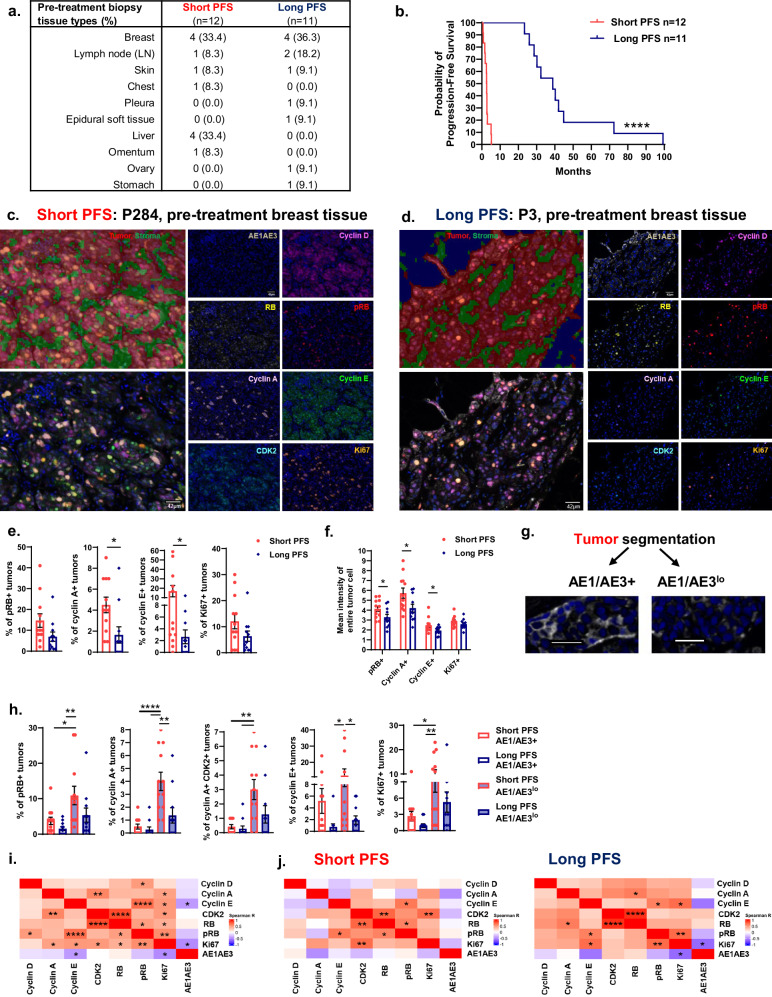
Low pre-treatment tumor levels of cyclin A or E associate with long PFS. **a** Summary of patient biopsy tissue types. **b** Significantly longer progression-free survival (PFS) of patients classified as long PFS (>23 months; *n* = 11) than short PFS (<6 months; *n* = 12). **c**, **d** Representative tissue segmentation and multispectral immunofluorescent (mIF) imaging of pre-treatment tissue from patients with short (**c**) or long (**d**) PFS. Scale bar: 42 μm. **e**, **f** Quantification of percentages (**e**) or mean intensities (**f**) of cell cycle proteins within the overall tumor segmentation. **g** Additional analyses of tumor segmentation based on AE1/AE3^+^ or AE1/AE3^lo^ expression. Scale bar: 42 μm. **h** Quantification of cell cycle protein percentages among AE1/AE3^+^ or AE1/AE3^lo^ tumors. **i**, **j** Correlations of cell cycle proteins with AE1/AE3 in pre-treatment tissue of the combined cohort (**i**) or based on patient stratification of PFS (**j**). Data are represented as mean ± SEM. Significance was determined by log rank test (**b**), unpaired t test with Welch’s correction (**e**, **f**), Holm-Bonferroni correction for pre-planned multiple comparisons (**h**) or Spearman correlation (**i**, **j**; *n* ≥ 11 patients/group). *p < 0.05; **p < 0.01; ***p < 0.001; ****p < 0.0001.

A sub-cohort of patients with short or long PFS (Fig. 2a, b) was selected for mIF imaging to analyze baseline cell cycle proteins among pre-CDK4/6i tissue that was segmented as tumor or stroma (Fig. 2c, d). In addition to cyclin D1, a binding partner of CDK4/6, we designed a panel that focused on proteins downstream of CDK4/6 signaling including phosphorylated RB (pRB), cyclin A, cyclin E, and CDK2^32^. Cell cycle proteins were robustly detected among tumor tissue (Supplementary Fig. 2a). Patients with long PFS had significant decreases in pre-treatment tumor cell cycle protein abundance of cyclin A and E (Fig. 2e). Cell cycle protein expression was also assessed as cellular mean intensity and significant declines in pRB, cyclin A and E mean intensities were observed in pre-treatment tumors of patients with long PFS (Fig. 2f and Supplementary Fig. 2b). The overall tumor segmentation was further analyzed based on differential expression of the epithelial pan-cytokeratin marker AE1/AE3 (Fig. 2g). While AE1/AE3^+^ tumor cells had significantly higher AE1/AE3 mean intensity as expected, AE1/AE3^lo^ tumors were more abundant than AE1/AE3^+^ tumors in pre-CDK4/6i tissue of patients with long or short PFS (Supplementary Fig. 2c, d). Average tumor cell or nuclear size did not differ based on AE1/AE3 expression (Supplementary Fig. 2e, f). However, continuous tumor island area of AE1/AE3^+^ tumors in patients with short PFS were significantly larger than AE1/AE3^lo^ tumor island area observed in patients with long or short PFS (Supplementary Fig. 2g). Overall pre-treatment tumor island area was similar among patients (Supplementary Fig. 2h). Cell cycle protein expression of AE1/AE3^lo^ tumor cells were comparable to the overall segmented tumor tissue (Fig. 2h and Supplementary Fig. 2i). AE1/AE3^lo^ tumor cells of patients with short PFS exhibited greater abundance of pRB, cyclin A, cyclin A-CDK2, or Ki67 in comparison to AE1/AE3^+^ tumor cells of patients with long or short PFS. Interestingly, AE1/AE3^+^ tumors negatively correlated with cyclin E or Ki67 across the combined patient cohort (Fig. 2i). Pre-treatment tumor cell cycle correlations were then independently compared among patients with short or long PFS (Fig. 2j). Pre-treatment Ki67^+^ tumors significantly correlated with CDK2 levels in patients with short PFS and pRB or cyclin E in patients with long PFS. Nuclear and cytosolic mean intensities of cyclins and CDK2 demonstrated high nuclear to cytosol ratios as anticipated for nuclear activity (Supplementary Fig. 2j). Finally, we examined the stroma which displayed a lesser extent of cell cycle proteins as anticipated. While RB mainly comprised the stroma, no significant differences were observed in baseline stromal cell cycle profiles of patients with long or short PFS (Supplementary Fig. 2k, l). Overall, our results demonstrate that AE1/AE3^lo^ tumor cells are abundant among pre-treatment biopsies and express greater levels of cyclin A or E in patients with short PFS. Furthermore, AE1/AE3^+^ tumors have larger continuous tumor island area than AE1/AE3^lo^ tumors of patients with short PFS and express fewer regulators of proliferation including pRB and Ki67.

**Fig. 3:**
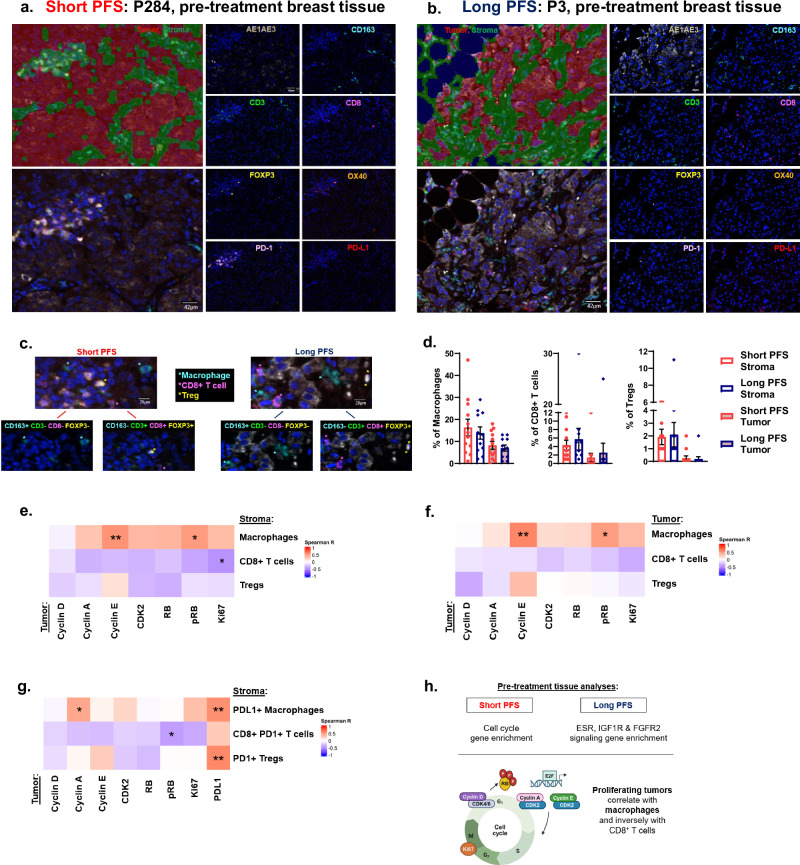
Pre-treatment cyclin E^+^ tumors directly correlate with macrophages while cyclin A^+^ tumors correlate with PD-L1^+^ macrophages. **a**, **b** Representative tissue segmentation and mIF imaging of pre-treatment tissue from patients with short (**a**) or long (**b**) PFS. Scale bar: 42 μm. **c** Representative images of detected immune cells. Scale bar: 20 μm. **d** Quantification of immune cell percentages. **e–g** Correlations of pre-treatment tumor cell cycle proteins with stromal (**e**), intratumoral (**f**) or immune checkpoint molecule-expressing stromal (**g**) immune cells. **h** Summary of pre-treatment tissue analyses. Figure created with BioRender.com. Data are represented as mean ± SEM. Significance was determined by Holm-Bonferroni correction for pre-planned multiple comparisons (**d**) or Spearman correlation (**e**–**g**; *n* ≥ 11 patients/group). *p < 0.05; **p < 0.01.

We designed an independent immune panel to examine T cell and macrophage presence in the pre-treatment TME (Fig. 3a–c). CD8^+^ T cells are instrumental for antitumor activity and evidence demonstrates that CDK4/6i-based therapy may activate CD8^+^ T cells while reducing immunosuppressive Tregs^15,16,19^. Macrophages largely compose breast tumors, inhabit potential metastatic sites and negatively associate with breast cancer patient survival^14,33^ but their roles during CDK4/6i therapy remain unclear. Macrophages (CD163^+^ CD3^-^ FOXP3^-^) were frequently detected within stroma and tumor tissue while CD8^+^ T cells (CD3^+^ CD8^+^ FOXP3^-^) and Tregs (CD3^+^ CD8^-^ FOXP3^+^) were commonly detected in the stroma with no statistical differences observed between stromal and tumor immune cell percentages (Fig. 3d). Since multiple tissue types of pre-treatment biopsies were examined (Fig. 2a), immune cell variability was expected due to tissue-specific preferences for immune cell inhabitance and recruitment^34,35^. To account for tissue-specific immune effects, we separately analyzed pre-CDK4/6i breast tissue as a common biopsy site to further study immune cell abundance and spatial proximity to AE1/AE3^+^ tumor cells (Supplementary Fig. 3a–d). High but insignificant CD8^+^ T cell numbers were particularly observed among AE1/AE3^+^ tumor cells within pre-treatment breast tissue of patients with long PFS (*n* = 4/group). Although limited in the number of common biopsy sites, our imaging analyses suggest tissue-specific effects of immune cell-AE1/AE3^+^ tumor cell localization in addition to immune cell frequency among the pre-CDK4/6i TME.

T cells and macrophages were then assessed for immune checkpoint molecule expression involving the PD-1-PD-L1 axis. PD-1 is upregulated upon T cell activation and chronic PD-1 signaling contributes to T cell exhaustion^36^. Macrophages and tumor cells may express PD-L1, the ligand for PD-1 that reinforces negative regulation of T cell activation^37^. Few PD-L1^+^ tumors were detected (Supplementary Fig. 3e). Low percentages of PD-L1^+^ macrophages, CD8^+^ PD-1^+^ T cells and PD-1^+^ Tregs were also observed in pre-CDK4/6i stroma or tumor tissue (Supplementary Fig. 3f). Independent of the PD-1-PD-L1 pathway, OX40 was included in our panel as a potential T cell activation marker^28^ and very few OX40^+^ T cells were observed (Supplementary Fig. 3g).

Correlations were performed to assess pre-treatment cell cycle-immune associations among similar regions-of-interest stained independently for cell cycle and immune markers among the combined patient cohort. Tumor cell cycle proteins largely correlated with stromal or intratumoral macrophages while inversely correlating with T cell detection (Fig. 3e, f). Ki67^+^ tumors negatively correlated with stromal CD8^+^ T cells. Cyclin E^+^ or pRB^+^ tumors directly correlated with macrophages. Cyclin E levels in the stroma also correlated with stromal macrophages and to a lesser extent Tregs (*p* = 0.067; Supplementary Fig. 3h). Finally, we examined correlations of PD-L1^+^ tumors with stromal immune cells expressing immune checkpoint molecules (Fig. 3g). PD-L1^+^ tumors directly correlated with PD-L1^+^ macrophages and PD-1^+^ Tregs. Cyclin A^+^ tumors also directly correlated with PD-L1^+^ macrophages while pRB^+^ tumors negatively correlated with CD8^+^ PD-1^+^ T cells. Our studies suggest that tumor cell cycle determinants and PD-L1 expression associate with immune cell frequency in the pre-CDK4/6i TME (Fig. 3h).

### Alterations in tumor biology and immune cell profiles during therapy

**Fig. 4:**
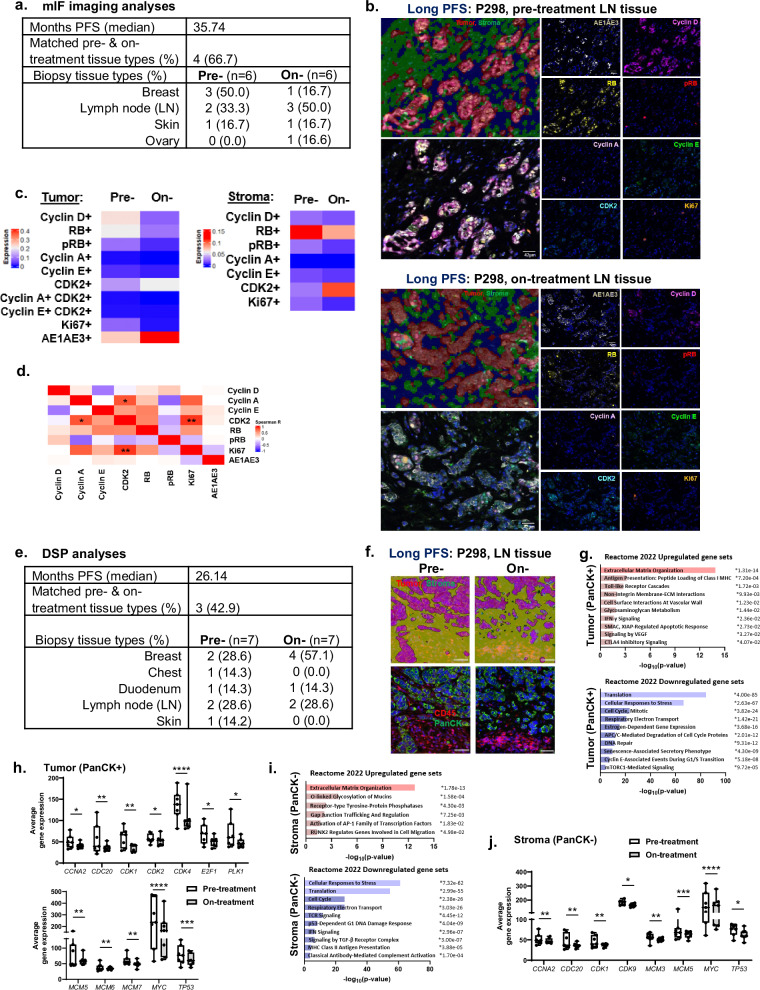
Low levels of pRB or Ki67 observed in CDK4/6i-treated tumors and stroma across tissue types of patients with long PFS. **a** Summary of pre- and on-treatment biopsy tissue types for mIF imaging analyses. **b** Representative tissue segmentation and mIF imaging of pre- (top) and on-treatment (bottom) tissue from patients with long PFS. Scale bar: 42μm. **c** Heatmaps of average cell cycle protein expression during CDK4/6 inhibitor (CDK4/6i)-based treatment within tumors (left) or stroma (right) in comparison to pre-treatment levels. **d** Correlations of cell cycle proteins with AE1/AE3 during treatment. **e** Summary of pre- and on-treatment biopsy tissue types for DSP analyses. **f** Representative tissue segmentation, pan-cytokeratin (PanCK) and CD45 staining of pre- or on-treatment tissue. Scale bar: 50 μm. **g** Enrichr plots of gene sets upregulated (top) or downregulated (bottom) among treated tumor tissue. **h** Reduced cell cycle transcripts in treated tumor tissue. **i** Enrichr plots of gene sets upregulated (top) or downregulated (bottom) among treated stroma. **j** Cell cycle gene expression also decreases among on-treatment stroma. Data are represented as mean ± SEM. Significance was determined by unpaired t test with Welch’s correction (**c**), Spearman correlation (**d**), Fisher’s exact test (**g**, **i**) or Wald test (**h**, **j**; *n* ≥ 6 patients/group). *p < 0.05; **p < 0.01; ***p < 0.001; ****p < 0.0001.

To gain insights into the tumor-intrinsic response to first-line CDK4/6i-based therapy, we focused on a small cohort of patients with long PFS (Fig. 4a). Pre-treatment and on-treatment biopsies were examined for tumor morphology and transcriptomic analyses. mIF images revealed intriguing tumor morphology changes involving AE1/AE3 expansion during therapy (Fig. 4b and Supplementary Fig. 4a). While AE1/AE3^lo^ tumors were abundant in pre-CDK4/6i tissue, similar percentages of AE1/AE3^+^ and AE1/AE3^lo^ tumors were observed among on-treatment biopsies (Supplementary Fig. 4b). AE1/AE3 mean intensity, tumor cell size, nuclear size (*p* = 0.14) and continuous tumor island area (*p* = 0.23) were similar among pre-treated and treated AE1/AE3^+^ tumors (*n* = 6/group; Supplementary Fig. 4c–f). During therapy, AE1/AE3^+^ tumor island area was significantly larger than AE1/AE3^lo^ tumors. Overall continuous tumor island area (*p* = 0.26) was similar while individual assessments of AE1/AE3^+^ tumor island area suggested expanding tumor morphology within treated tissue (Supplementary Fig. 4g, h). Interestingly, individual assessments also displayed inverse patterns of tumor PD-L1 expression with AE1/AE3 expansion. Differential gene expression among pre- and on-treatment tissue was assessed by targeted RNA-seq (Supplementary Fig. 4i, j). Gene expression of signaling pathways associated with anti-apoptosis (*BCL2*), FGF (*FGFR2/3*), *STAT6*, and the extracellular matrix enzyme *MMP9* were significantly reduced on-treatment. Whereas gene expression associated with AP-1 (*FOS*), MAPK (*MAPK10*), *PTGS2*, select growth factors (*ANGPT1*, *EGR1*, *TDGF1*, *TGFB2*), and tissue structure (*COL4A6*, *COL9A3*, *KRT16*) were significantly higher in treated tissue and may contribute to on-treatment tissue morphology.

Alteration of cell cycle protein dynamics during treatment was examined by mIF imaging in comparison to pre-treatment tissue (Fig. 4b). Based on an averaged assessment of protein markers (*n* = 6/group; Fig. 4c), low levels of cyclin D, RB, pRB or Ki67 within treated tumors (*p* = 0.13, 0.22, 0.17, 0.11) and pRB or Ki67 in treated stroma (*p* = 0.16, 0.13) were observed. On-treatment tumor expression of cyclin A or E remained low in this small cohort of patients with long PFS. Treated tumors displayed high AE1/AE3 levels (*p* = 0.18), supporting mIF images of tumor morphology expansion (Fig. 4b and Supplementary Fig. 4a). Interestingly, CDK2 levels within treated tumors significantly correlated with cyclin A or Ki67 (Fig. 4d).

To further assess tumor-intrinsic responses to first-line CDK4/6i, a distinct patient cohort was utilized (Fig. 4e). Tumor tissue was distinguished from stroma based on pan-cytokeratin expression within pre- and on-treatment biopsies for digital spatial profiling (DSP) and transcriptomic analyses (Fig. 4f). Within treated tumors, gene enrichment for ECM organization, MHC class I antigen presentation pathways and toll-like receptor (TLR) signaling were detected based on the Reactome 2022 pathway database (Fig. 4g). Downregulated gene sets among treated tumors involve translation, cellular responses to stress and cell cycle. Multiple cell cycle genes (*CCNA2*, *CDC20*, *CDK1/2/4*, *E2F1*, *PLK1*), replication factors (*MCM5/6/7*), *MYC*, and *TP53* were significantly reduced in treated tumors (Fig. 4h). Few upregulated gene pathways were observed within on-treatment stroma and mainly involve ECM (Fig. 4i). Similar to treated tumors, cellular responses to stress, translation and cell cycle gene sets were downregulated among on-treatment stroma with significantly reduced cell cycle genes (*CCNA2*, *CDC20*, *CDK1/9*), replication factors (*MCM3/5*), *MYC*, and *TP53* transcripts (Fig. 4j). Collectively from two distinct CDK4/6i-treated patient cohorts, our imaging and transcriptomic analyses of the TME revealed a reduction in cell cycle expression within tumor and stroma tissue along with ECM gene enrichment and tumor morphological changes during CDK4/6i therapy.

**Fig. 5:**
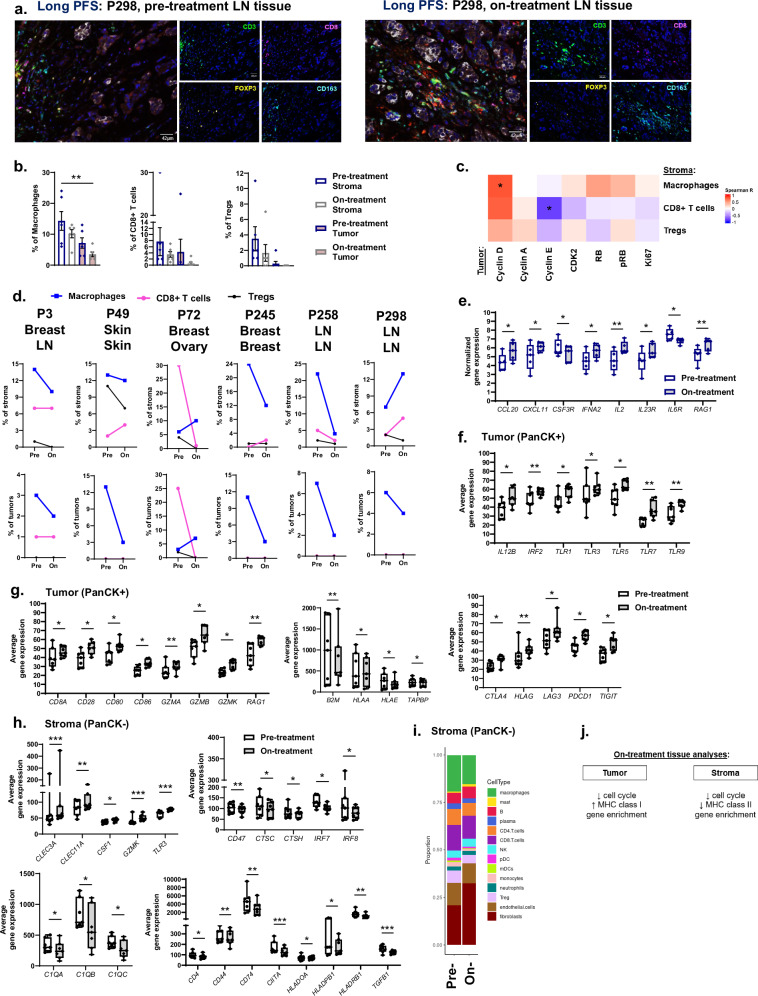
Gene enrichment associated with T cell activation emerge during CDK4/6i treatment. **a** Representative mIF imaging of pre- (left) and on-treatment (right) tissue from patients with long PFS. Scale bar: 42 μm. **b** Quantification of immune cell percentages within pre- and on-treatment tissue. **c** Correlations of CDK4/6 inhibitor (CDK4/6i)-treated tumor cell cycle proteins with stromal immune cells. **d** Individual assessments of stromal (top) or intratumoral (bottom) immune cell percentages from matched pre- (listed first) and on-treatment tissue. **e** Differential immune gene expression during therapy among patients with long PFS. **f**, **g** DSP analyses of treated tumor tissue revealed increased immune gene expression of toll-like receptors (**f**), co-stimulatory molecules involving the MHC class I pathway and immune checkpoints (**g**). **h** DSP analyses of on-treatment stroma indicated increased expression of select immune-stimulatory transcripts while reduced gene expression of complement and co-stimulatory molecules involving the MHC class II pathway. **i** Deconvolution assay to predict proportions of stromal immune cells. **j** Summary of on-treatment tissue analyses. Data are represented as mean ± SEM. Significance was determined by Holm-Bonferroni correction for pre-planned multiple comparisons (**b**), Spearman correlation (**c**), Wald test (**e**–**h**), or unpaired t test with Welch’s correction (**i**; *n* ≥ 5 patients/group). *p < 0.05; **p < 0.01; ***p < 0.001.

We then evaluated the effects of CDK4/6i treatment on T cell and macrophage abundance within the TME compared to pre-treatment tissue using mIF imaging (Fig. 5a and Supplementary Fig. 5a). Although low macrophage percentages were observed among stromal (*p* = 0.25) and tumor (*p* = 0.19) tissue during therapy, macrophage and T cell detection were similar to pre-treatment tissue (*n* = 6/group; Fig. 5b). Correlations of tumor cell cycle proteins during therapy demonstrated that cyclin D^+^ tumors directly correlated with stromal macrophages and to a lesser extent CD8^+^ T cells (*p* = 0.068; Fig. 5c). Cyclin E^+^ tumors inversely correlated with stromal CD8^+^ T cells while modestly trending with intratumoral macrophages (*p* = 0.19; Supplementary Fig. 5b). Within the stroma, modest correlation trends of cyclin A with macrophages (*p* = 0.14) and pRB or Ki67 with Tregs (*p* = 0.09, 0.18; Supplementary Fig. 5c) were observed. Spatial immune cell analyses and the number of neighboring immune cells to AE1/AE3^+^ tumors during treatment were not altered (Supplementary Fig. 5d, e). Variability in immune cell detection was expected since on-treatment biopsies were obtained from multiple tissue types and may not have been identical to the corresponding pre-treatment biopsy tissue type (Fig. 4a). To account for tissue type differences, we individually assessed immune cell profiles during treatment (Fig. 5d). Individual patterns of less macrophages and stromal Tregs were commonly observed in response to CDK4/6i. Whereas high stromal CD8^+^ T cells during treatment were observed in patients with matched pre- and on-treatment tissue types (P49, P245 and P298), suggesting tissue-specific effects of the CD8^+^ T cell response.

Immune alterations during therapy were further examined by transcriptomics and mIF imaging analyses of the PD-1-PD-L1 axis. Highly expressed immune genes during therapy involve select chemokines (*CCL20*, *CXCL11*), cytokines (*IFNA*, *IL2*, *IL23R*) and the recombinant enzyme *RAG1* (Fig. 5e). RAG1, IL-2 and CXCL11 expression are especially important for T cell activation, expansion and chemotaxis^28,38^, suggesting emergence of gene enrichment for T cell activation during CDK4/6i therapy. Whereas *CSF3* and *IL6R* transcripts often associate with immunosuppressive granulocyte responses^37^ and significantly decreased during treatment. Low PD-L1 levels were detected on tumor cells or macrophages while low PD-1 expression was observed on T cells during treatment (Supplementary Fig. 5f, g). Modest correlation trends of stromal PD-L1^+^ macrophages (*p* = 0.12) with Ki67^+^ or PD-L1^+^ tumors were observed (Supplementary Fig. 5h). Based on individual assessments, less stromal PD-1^+^ T cells or PD-L1^+^ macrophages were commonly observed during treatment across tissue types (Supplementary Fig. 5i). Although limited in sample size, our imaging results collectively suggest low expression of cell cycle proteins and the immunosuppressive PD-1-PD-L1 axis during CDK4/6i treatment.

Additional tumor-extrinsic responses to CDK4/6i were assessed with DSP and transcriptomic analyses. Immune-stimulatory genes highly abundant within tumors during therapy involve TLRs (*TLR1/3/5/7/9*), *IL12B*, co-stimulatory molecules (*CD8A*, *CD28*, *CD80*, *CD86*), cytotoxic mediators (*GZMA/B/K*) and components of the MHC class I antigen pathway (*B2M*, *HLA-A/E*, *RAG1*, *TAPBP*; Fig. 5f, g). CDKs commonly regulate transcriptional activation downstream of TLR signaling during innate immune responses^39^. Interestingly, immune checkpoint molecule (*CTLA4*, *LAG3*, *PDCD1*, *TIGIT*) and inhibitory molecule (*HLA-G*) gene expression were also increased within treated tumors and may be a consequence of immune activation^28^. Immune-stimulatory genes involving TLR- or CLEC-mediated recognition (*TLR3*, *CLEC3A/11* *A*), macrophage recruitment (*CSF1*) and cytotoxicity (*GZMK*) were increased within stroma during therapy (Fig. 5h). However, multiple immune gene sets including TCR signaling, MHC class II antigen presentation and complement pathways were downregulated (Fig. 4i). Stromal immune-stimulatory genes reduced during therapy involve complement (*C1QA/B/C*), cathepsin (*CTSC/H*), IRFs (*IRF7/8*), components of the MHC class II antigen pathway (*CD74*, *CIITA*, *HLA-DOA/DPB1/DRB1*), and costimulatory molecules (*CD4*, *CD44*) along with immunoregulatory molecules *TGFB1* and *CD47* for anti-phagocytosis (Fig. 5h). A deconvolution analysis was performed to predict the proportion of stromal immune cells based on gene expression (Fig. 5i). Based on an averaged assessment (*n* = 7/group), low proportions of monocytes, a precursor of macrophages^33^, were observed during treatment (*p* = 0.057). Overall, our DSP analyses indicated that treated tumors display gene enrichment for MHC class I pathways, immune stimulation characteristic of CD8^+^ T cell responses and granzyme-mediated cytotoxicity (Fig. 5j). In contrast, treated stroma displayed reduced gene expression of MHC class II pathways, immune stimulation characteristic of CD4^+^ T cell helper responses and complement-mediated cytotoxicity.

## Discussion

Administration of CDK4/6i as a targeted therapy in combination with endocrine therapy has prolonged PFS of metastatic HR + /HER2- breast cancer and recently ribociclib and abemaciclib gained approval in combination with aromatase inhibitors for high-risk early-stage breast cancer^1,4,40^. However, the efficacy of this therapeutic combination remains limited and incurable^6^. Here, we focused on a “real-world” clinical assessment to identify baseline tumor-intrinsic and -extrinsic indicators of a patient cohort with strong differential responses to standard-of-care CDK4/6i-based therapy. Transcriptomic analyses demonstrated pre-treatment gene enrichment for growth factor and immune signaling with a corresponding reduction of cell cycle gene expression among patients with long PFS. Long PFS on therapy associates with low pre-treatment tumor levels of cyclin A or E and this finding is well-supported by pre-clinical and clinical evidence in which cyclin E overexpression underlies CDK4/6i resistance by promoting cell cycle progression^9–11,41^. Intratumoral cell cycle proteins commonly correlate with macrophage accumulation and Ki67^+^ tumors negatively correlate with CD8^+^ T cell detection.

We performed a binary selection of our clinical trial cohort to dichotomize CDK4/6i response which adds unique clinical significance of defining long-term response in comparison to recent “real-world” studies evaluating broad clinical response cohorts^26,27^. However, we recognize that multiple clinical characteristics such as prior treatment, visceral metastatic disease or type of endocrine therapy may skew the short PFS cohort^11^. To alleviate the confounding variable of prior treatment, we analyzed differential gene expression among pre-treatment biopsies of the PFS-stratified cohorts that received CDK4/6i first-line. A deeper evaluation of prior therapy effects, including mechanisms underlying endocrine therapy resistance, and tissue-specific effects of metastatic presentation on CDK4/6i response remain to be determined.

We designed a combinational molecular approach to study cell cycle biology and immune cell abundance within the TME of pre- and on-treatment tissue. Protein-based imaging offers a superior approach for phenotyping cellular markers by detecting subcellular marker localization, quantifying cell abundance among defined tissue segmentations that distinguish tumor from stroma, and studying cell interactions based on spatial location^42,43^. Unlike non-invasive peripheral measures, TME analyses are more informative for revealing changes in tumor morphology or tumor-immune spatial interactions. Our work applies to the diverse, “real-world” setting as we observed multiple biopsy tissue types of prevalent metastatic sites of HR + /HER2- breast cancer. As immune cells may display tissue-specific preferences of residence^34^ and metastatic sites may have disproportionate immune cell recruitment^35^, future work may require similar tissue analyses to validate immune cell detection.

Our imaging analyses recognize the impact of tumor-intrinsic cell cycle protein expression on corresponding tumor epithelial characteristics and morphology. Pre-treatment AE1/AE3^lo^ tumors of patients with short PFS are significantly more abundant and have smaller tumor island area than AE1/AE3^+^ tumors. Cyclin A is particularly higher in pre-treatment AE1/AE3^lo^ tumors of patients with short PFS while cyclin E is largely expressed regardless of AE1/AE3 expression. This suggests that pre-treatment tumors displaying less cytokeratin are a dominant tumor population to therapeutically target and may be more susceptible to cyclin A- or E-mediated resistance mechanisms. Whether this dominant tumor population displays mesenchymal characteristics such as vimentin or exists in a hybrid EMT state^30,37^ remains to be further examined. Understanding tumor cell cycle biology and epithelial properties is also of importance during CDK4/6i therapy as our imaging analyses revealed observations of AE1/AE3^+^ tumor cell expansion in patients with long PFS. Furthermore, an assessment of growth factor pathways in addition to estrogen receptor signaling that contribute to pre-treatment tumor growth and the expansion of AE1/AE3^+^ tumor cells on-treatment remain to be determined. Pre-treatment *IGF1R* and *FGFR2* transcripts were significantly higher in patients with long PFS, suggesting that such growth factor pathways may be more susceptible to targeting with CDK4/6i^3,8^ and demonstrate gene concordance with clinical findings of PALOMA-2^10,13^. However, the functional dynamics of IGF1R and FGFR2 expression during treatment remain to be evaluated.

The roles of tumor-intrinsic cyclin expression on crosstalk with surrounding immune cells remain unclear. Few studies have focused on immune cell-intrinsic cell cycle protein expression as regulators of immune cell proliferation in absence of the TME or tumor cell cycle biology^44,45^. A study elsewhere demonstrated that stromal fibroblast expression of cyclin D1 enhanced angiogenesis and macrophage recruitment within a preclinical mammary tumor model^46^. Our cell cycle-immune cell correlations of pre-treatment tissue indicate that tumor-intrinsic cell cycle proteins largely associate with stromal or intratumoral macrophage accumulation rather than T cells. Macrophages correlated with cyclin E^+^ tumors while PD-L1^+^ macrophages particularly correlated with cyclin A^+^ tumors. Furthermore, our cell cycle-immune cell correlations of treated tissue revealed that cyclin E^+^ tumors negatively correlated with stromal CD8^+^ T cells and suggest that tumor-intrinsic cyclin E may facilitate CD8^+^ T cell exclusion during treatment. The underlying mechanisms of CD8^+^ T cell exclusion, which may involve ECM protein barriers such as collagen^14^, and the functional modes of macrophage accumulation, which may range from immune stimulation to immune suppression involving PD-L1^33^, within the pre-CDK4/6i TME remain of further investigation.

We focused on the PD-1-PD-L1 axis as a potential resistance mechanism that may limit cytotoxicity of the endogenous CD8^+^ T cell response^28,36,37^. While low pre-treatment percentages of CD8^+^ PD-1^+^ T cells or PD-L1^+^ macrophages were detected, the abundance of immune-stimulatory receptors that facilitate effector activation of CD8^+^ T cells or tumor phagocytosis and antigen presentation by macrophages remain to be evaluated. Assessing ratios of immune-stimulatory to immunosuppressive receptor expression on CD8^+^ T cells or macrophages^33^ may provide functional insights for additional targeting of the immune response during CDK4/6i therapy. Our DSP analyses suggest that MHC class I pathway genes are prominent within treated tumors while MHC class II pathway genes are downregulated within stroma. Inhibiting exhaustion of MHC class I-mediated tumor antigen recognition while strengthening MHC class II-mediated antigen presentation may enhance long-term activation of endogenous anti-metastatic immune responses to prolong CDK4/6i efficacy. Regarding tumor-mediated immune exhaustion, pre-treatment tumors expressed low levels of PD-L1 consistent with other reports of ER+ breast cancer^47^. In studies elsewhere, preclinical tumor models suggested that palbociclib and particularly CDK4 inhibition increases tumor expression of PD-L1^48^. During a single time point on-treatment, we observed low PD-L1, cyclin D or pRB expression and AE1/AE3 expansion within tumors. Whether tumor PD-L1 levels increase over time due to chronic CDK4 inhibition and impede endogenous anti-metastatic immune responses during CDK4/6i treatment remain to be further examined. Additionally, tumor-intrinsic and -extrinsic assessments of post-treatment tissue are warranted for gaining insights into potential resistance mechanisms acquired during long-term CDK4/6i response.

Our imaging and transcriptomic assessments of the pre-treatment TME offer implications for selective therapeutic targeting based on prevalent cell cycle or immune cell indicators. In pre-CDK4/6i TMEs with high cell cycle expression, additional cell cycle-targeting approaches may be considered such as CDK2i to reduce cyclin A- or E-mediated cell cycle progression^3,27,49^. In pre-CDK4/6i TMEs with high macrophage accumulation or T cell infiltration, blockade of the PD-1-PD-L1 axis may enhance endogenous antitumor T cell activity^28,29,50^. While drug tolerability of immune checkpoint blockade varies depending on the CDK4/6i utilized^14,47^, subsequent administration of immune checkpoint blockade in temporal relation to T cell infiltration within CDK4/6i-treated tissue may be ideal for synergistic immune effects.

Overall, our findings underscore the importance of studying the “real-world” clinical TME to identify tumor-intrinsic cell cycle indicators and corresponding immune cell associations with CDK4/6i durability. Our work particularly recognizes the pre-treatment TME as a therapeutic opportunity for combinational precision-based approaches to improve CDK4/6i efficacy.

## Methods

### Patient data source and ethics

A retrospective and prospective clinical trial was developed to assess clinical features of metastatic HR + /HER2- breast cancer progression during CDK4/6i treatment (NCT04526587)^5^. Metastatic HR + /HER2- breast cancer patients ≥18 years of age and treated with a CDK4/6i may enroll. Inclusion criteria for this study involved diagnosis of metastatic estrogen receptor + /HER2- breast cancer and adjuvant treatment with a CDK4/6i during 2015-2024. All patients provided written informed consent and all clinical study documentation, data collection and research were performed in accordance with the guidelines and regulations of Roswell Park Comprehensive Cancer Center Institutional Review Board (protocol #I-571719) and the Declaration of Helsinki. As of January 2025, over 300 patients provided consent, with 155 pre-treatment tissue analysis results available from our clinical trial (Supplementary Table 1). For this study, a binary selection of patients was performed to dichotomize patient response to CDK4/6i and is described in the following Methods section. Pre- and on-treatment biopsies (Supplementary Table 2) were collected during standard-of-care procedures via the Roswell Park protocol #I-571719 and according to the Declaration of Helsinki with de-identified sample labeling.

### Patient selection based on PFS

For female patients with tissue analysis results, patient charts were reviewed to stratify PFS on CDK4/6i-based therapy to dichotomize patient response. In this study, PFS was defined as time from the administration of the first dose of CDK4/6i to evidence of scan-based or biopsy-proven disease progression as determined by the medical oncologist or death. Twenty-nine patients with short PFS (defined as <6 months) and sixty-eight patients with long PFS (defined as >23 months) were selected as extremely unfavorable or favorable responses (Table 1). Long or short PFS stratification is based on real-world evidence of CDK4/6i therapeutic efficacy in multiple clinical trials^5,20–25^. For patients with long PFS that currently remain on treatment, PFS is measured based on the last patient follow-up as of January 6, 2025. Termination of CDK4/6i-based treatment for patients with long PFS mainly occurred due to progression and few occurrences of excessive toxicity or death on therapy were observed. For patients with short PFS, termination of treatment only occurred due to progression on therapy. Electronic medical records via a REDCap database were examined to collect patient demographics, clinical and pathological features as displayed in Table 1.

### Pathological tissue assessment and sectioning

Patient surgical pathology case slides were reviewed by a pathologist (A.K.W.) for adequate tissue and high tumor cellularity. Formalin-fixed paraffin-embedded (FFPE) blocks of pre- and on-treatment biopsy tissue were sectioned at 4 µm and placed on charged slides. Slides were dried at 37 ^o^C and utilized for imaging or targeted RNA-seq analyses.

### Targeted RNA-seq analyses

Clinical pre- and on-treatment tissue slides were submitted to HTG Molecular Diagnostics, Inc for targeted RNA-seq analyses based on the HTG EdgeSeq Oncology Biomarker panel^10,11^. Four independent runs were performed and raw data was corrected for batch effect using the ComBat-seq program^51^. Data was then normalized using the edgeR program^52^, reported as mean gene expression and utilized for further analyses. Gene set enrichment analysis (GSEA) in the GSEA software (version 4.2.1) was utilized as an unbiased approach of pre-treatment gene expression based on the Molecular Signatures Database (MSigDB). Differential gene expression analyses were performed using ‘DESeq2’ program^53^ (version 1.38.3) in RStudio software (version 4.2.0). Significant differential genes were uploaded into Enrichr^54^ and gene sets were displayed as Enrichr plots based on gene enrichment with the Reactome 2024 pathway database. Select differential transcripts were displayed as box-plots with individual points, whiskers depicting minimum to maximum values and lines denoting mean gene expression. The impact of pre-treatment gene expression on association with PFS was assessed with univariate Cox proportional-hazard regression. Transcript correlations were displayed as correlation heatmaps with the ‘ComplexHeatmap’ program^55^ in RStudio software (version 4.3.2).

### mIF staining and imaging analyses

Clinical pre- and on-treatment tissue slides were baked at 60 ^o^C for at least an hour and then deparaffinized in Leica Biosystems BOND Dewax solution (AR9222) within the BOND RX^m^ Research Stainer (Leica Biosystems). Staining for multispectral immunofluorescent (mIF) imaging analyses was performed with the AKOYA Biosciences reagent kit (NEL821001KT) and entailed serial repetitions of epitope retrieval with ER1 (citrate buffer pH 6, Leica Biosystems, AR996), addition of blocking buffer (AKOYA Biosciences) prior to sequential incubation with the primary antibody (specified below per panel) for the biomarker of interest, HRP secondary antibody (AKOYA Biosciences or Leica Biosystems, PV6119/PV114) and Opal Fluorophore (AKOYA Biosciences) for signal amplification and lastly antibody stripping. Primary antibodies for the cell cycle mIF panel included: cyclin D (ThermoFisher, MA1-39546), RB (Cell Signaling Technology, 9309), pRB (Cell Signaling Technology, 8516), cyclin A (Abcam, ab32386), cyclin E (Abcam, ab33911), CDK2 (Cell Signaling Technology, 18048), Ki67 (Abcam, ab16667), and AE1/AE3 (Dako, M3515). Primary antibodies for the immune mIF panel included: CD3 (Abcam, ab16669), CD8 (Dako, M7103), FOXP3 (Abcam, ab20034), CD163 (Leica Biosystems, CD163-L-U), PD-1 (Cell Signaling Technology, 43248), PD-L1 (Cell Signaling Technology, 13684), OX40 (Cell Signaling Technology, 61637), and AE1/AE3 (Dako, M3515). Following mIF staining, slides were manually stained with DAPI (AKOYA Biosciences) for nuclear detection and cover slipped with Diamond antifade mountant (Invitrogen, P36961).

Stained slides were initially scanned in an unmixed view within the PhenoImager^TM^ HT (AKOYA Biosciences). Selection of representative regions-of-interest (ROIs) were guided by a pathologist (A.K.W.). Similar ROIs were selected for the independently stained cell cycle and immune mIF panels to compare correlations of cell cycle and immune cell effects. ROIs were acquired by rescanning the stained slides. Phenotyping of the cell cycle or immune mIF panels was performed with the AKOYA Biosciences inForm software (version 2.6.0) and in a blinded manner regarding patient PFS stratification. A representative unmixed ROI from each patient was selected for the inForm software training set to train tissue segmentation, cellular segmentation and biomarker phenotyping. Phenotyping of each biomarker was achieved using a unique algorithm that utilizes machine learning for selection of biomarker positive or biomarker negative cells. Each biomarker algorithm was batch applied for all ROIs included for imaging analyses and quality control of resultant phenotyping batch assessments were observed.

Quantification of phenotyping data including cellular percentages, counts and proximity to AE1/AE3^+^ tumors was performed with ‘phenoptrReports’ program in RStudio software (version 4.2.2). QuPath program^56^ (version 0.5.1) was utilized to quantify continuous tumor island area. Phenotyping data was displayed as correlation heatmaps or indicated average protein expression heatmaps with the ‘ComplexHeatmap’ program^55^ in RStudio software (version 4.3.2).

### Digital spatial profiling (DSP) analyses

Clinical pre- and on-treatment tissue was prepared on Leica Apex BOND superior adhesive slides (Leica Biosystems, 3800040) and baked at 60 ^o^C for at least an hour. The BOND RX^m^ Research Stainer (Leica Biosystems) was prepared according to the GeoMx^TM^ DSP Automated Slide Preparation User Manual (MAN-10151-02). Slides were deparaffinized in Leica Biosystems BOND Dewax solution (AR9222) and ER2 (Tris-EDTA buffer pH 9, Leica Biosystems, AR9640) was utilized for epitope retrieval. A RNA-binding in situ hybridization (ISH) process with the human GeoMx Whole Transcriptome Atlas Probe Mix (NanoString Technologies, 121410104) was performed overnight. Slides were then stained with the human GeoMx Solid Tumor TME Morphology Marker kit (NanoString Technologies, 121300320) and a master mix composed of antibody-fluorophore complexes that bind to pan-cytokeratin or CD45 and a nuclear stain (SYTO 13).

Probed slides were scanned within the GeoMx^TM^ Digital Spatial Profiler (NanoString Technologies). ROI selection was guided by a pathologist (A.K.W.) and segmentation for pan-cytokeratin positive tissue was performed to distinguish tumor (PanCK^+^) from stroma (PanCK^-^). ROIs were then acquired and UV light was administered to expose the photocleavable oligonucleotides coupled to the RNA probes among the segmented ROIs. Released oligos were collected separately among PanCK^+^ or PanCK^-^ aspirates. The resultant libraries were sequenced using the Illumina platform. Sequencing files were processed to digital counts using the GeoMx^TM^ NGS pipeline. For quality control and data normalization, NanoString Technologies’ GeomxTools Bioconductor package^57^ (version 3.10.0) was utilized.

Differential gene expression analyses were performed using ‘DESeq2’ program^53^ (version 1.38.3) in RStudio software (version 4.2.0). Significant differential genes were uploaded into Enrichr^54^ and gene sets were displayed as Enrichr plots based on gene enrichment with the Reactome 2022 pathway database. Select differential transcripts were displayed as box-plots with individual points, whiskers depicting minimum to maximum values and lines denoting mean gene expression. Stromal immune cell deconvolution was analyzed with GeomxTools ‘Spatial Decon’ program^57^ (version 1.16.0) in RStudio software (version 4.2.0).

### Statistical methods

Data was analyzed with Prism software (version 10.2.2) for 2-group comparisons using unpaired t test with Welch’s correction (unequal variances) and for log rank tests to assess PFS displayed in Kaplan-Meier curves. Spearman correlations, Holm-Bonferroni correction for pre-planned multiple comparisons involving 4-groups (assessments of stroma versus tumor or AE1/AE3^+^ versus AE1/AE3^lo^ tumors in pre- and on-treatment tissue), and univariate Cox proportional-hazard regression were analyzed with RStudio software (version 4.3.2). Wald tests within the ‘DESeq2’ program in RStudio software (version 4.2.0) determined significant differential gene expression. Fisher exact tests determined significant gene sets displayed in Enrichr plots. Data are depicted as mean ± SEM with asterisks noted in figures and p values described in legends. *P*-values < 0.05 were considered statistically significant. Two independent experiments with large biological replicates were performed.

## Supplementary information

Supplementary Information

## Data Availability

Data generated or analyzed during this study are included in this published article and are available from the corresponding authors upon reasonable request.
