# Supplementary material for: Baseline cell cycle and immune profiles indicate CDK4/6 inhibitor response in metastatic HR + /HER2- breast cancer
Source: NPJ Breast Cancer. 2025 Jun 12;11:54. doi: 10.1038/s41523-025-00767-2 (PMC12162882; doi:10.1038/s41523-025-00767-2)
Supplement: Supplementary file 1 — Supplementary Information [file 41523_2025_767_MOESM1_ESM.pdf]

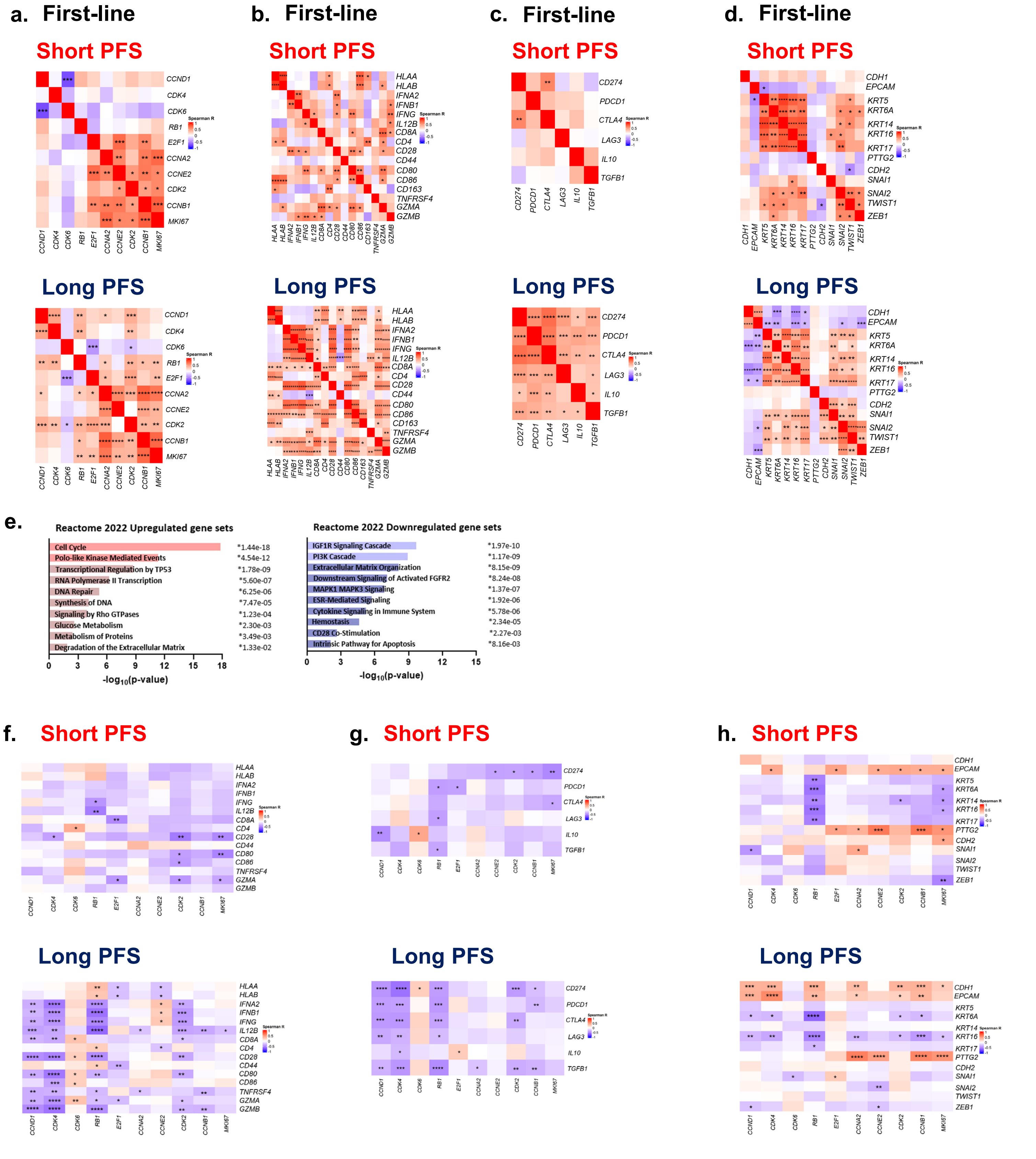

Supplementary Figure 1. Correlations of pre-treatment gene expression, Related to Figure 1. **a-d** Correlations of cell cycle (a), immune-stimulatory (b), immunosuppressive (c), or epithelial and mesenchymal (d) transcripts among pre-treatment tissue of first-line patients with short (top) or long (bottom) PFS. **e** Enrichr plots of pre-treatment gene sets upregulated (left) or downregulated (right) among the total cohort of patients with short PFS compared to patients with long PFS. **f-h** Correlations of cell cycle transcripts with immune-stimulatory (f), immunosuppressive (g), or epithelial and mesenchymal (h) gene expression in pre-treatment tissue of the total patient cohort with short (top) or long (bottom) PFS. Significance was determined by Spearman correlation (a-d, f-h) or Fisher's exact test (e; n ≥ 17 patients/group). *p<0.05; **p<0.01; ***p<0.001; ****p<0.0001.

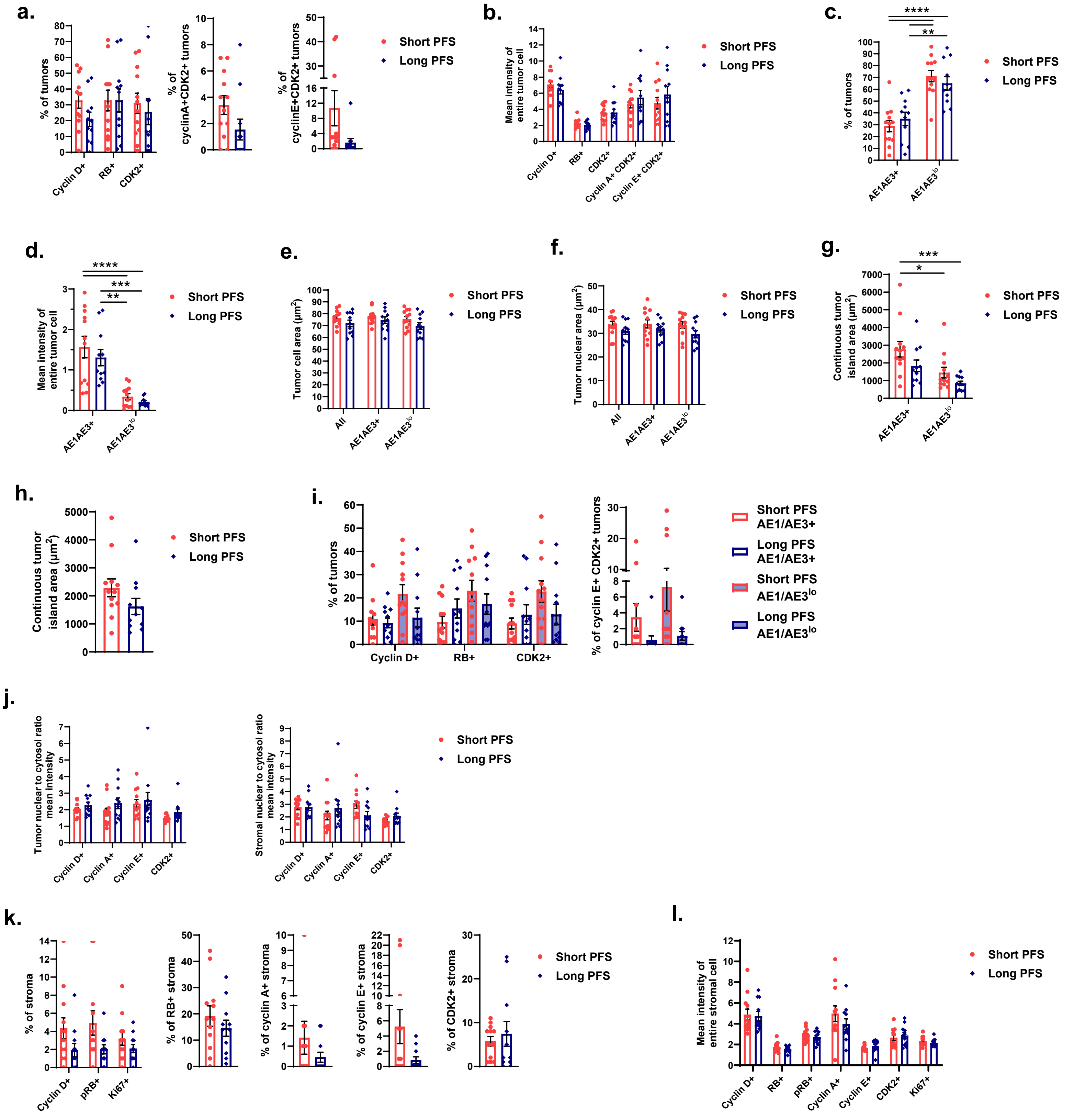

Supplementary Figure 2. Assessment of cell cycle proteins in relation to the overall tumor segmentation and cellular localization, Related to Figure 2. a, b Quantification of cell cycle protein percentages (a) and mean intensities (b) within the overall tumor segmentation. **c, d** Percentages (c) or mean intensities (d) of AE1/AE3 expression among AE1/AE3^{+} or AE1/AE3^{lo} tumors. AE1/AE3^{lo} tumors are most abundant among pre-treatment tissue. **e, f** Comparison of average tumor cell area (e) or nuclear area (f) among pre-treatment tissue. **g** Average AE1/AE3^{+} or AE1/AE3^{lo} tumor island area among pre-treatment patient biopsies. **h** Overall assessment of pre-treatment continuous tumor island area. **i** Percentages of cell cycle proteins within tumor cells based on differential AE1/AE3 expression. **j** Mean intensities of nuclear to cytosol ratios of cyclin or CDK2 expression within the overall tumor segmentation (left) or stroma (right). High nuclear to cytosol ratios of cyclins and CDK2 were expected for nuclear activity. **k, l** Stromal cell cycle protein percentages (k) and mean intensities (l). Data are represented as mean ± SEM. Significance was determined by unpaired t test with Welch's correction (a, b, e, f, h, j-l) or Holm-Bonferroni correction for pre-planned multiple comparisons (c, d, g, i; $n \geq 11$ patients/group).

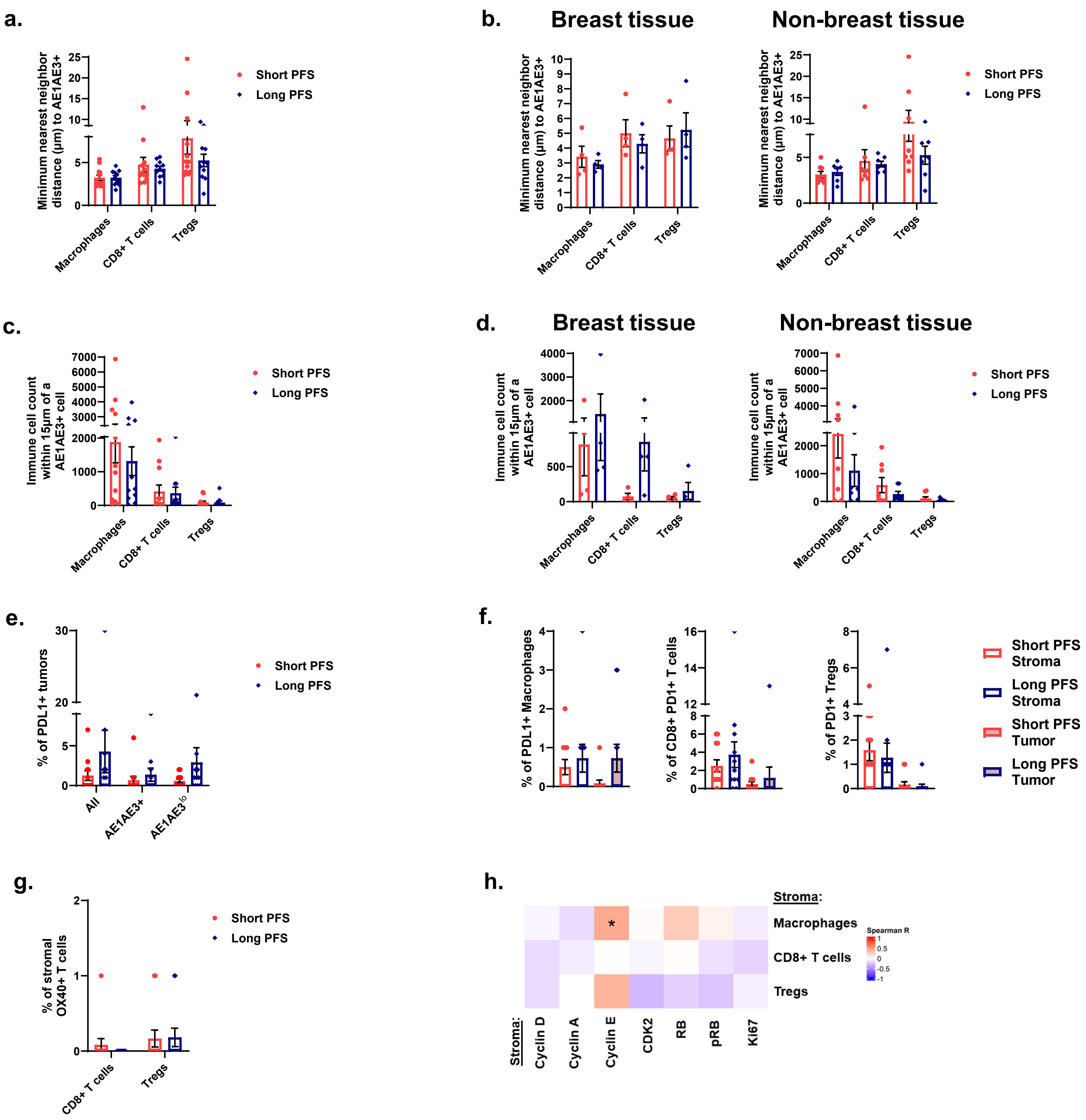

Supplementary Figure 3. Pre-treatment breast tissue of patients with long PFS display high CD8^{+} T cell numbers in proximity to AE1/AE3^{+} tumors, Related to Figure 3. a, b Quantification of immune cell proximity to a neighboring AE1/AE3^{+} tumor cell within pre-treatment tissue (a). Breast or non-breast pre-treatment tissue was separately analyzed for tissue-specific effects (b). **c, d** Quantification of immune cell count within a neighboring AE1/AE3^{+} tumor cell among pre-treatment tissue (c). Breast or non-breast pre-treatment tissue was separately analyzed for tissue-specific effects (d). **e, f** Percentages of tumors (e) and immune cells (f) with immune checkpoint molecule expression. **g** Very few percentages of OX40^{+} T cells detected in pre-treatment tissue. **h** Correlations of pre-treatment cell cycle proteins and immune cell detection within stroma. Data are represented as mean ± SEM. Significance was determined by unpaired t test with Welch's correction (a-e, g), Holm-Bonferroni correction for pre-planned multiple comparisons (f) or Spearman correlation (h; n ≥ 11 patients/group). *p<0.05.

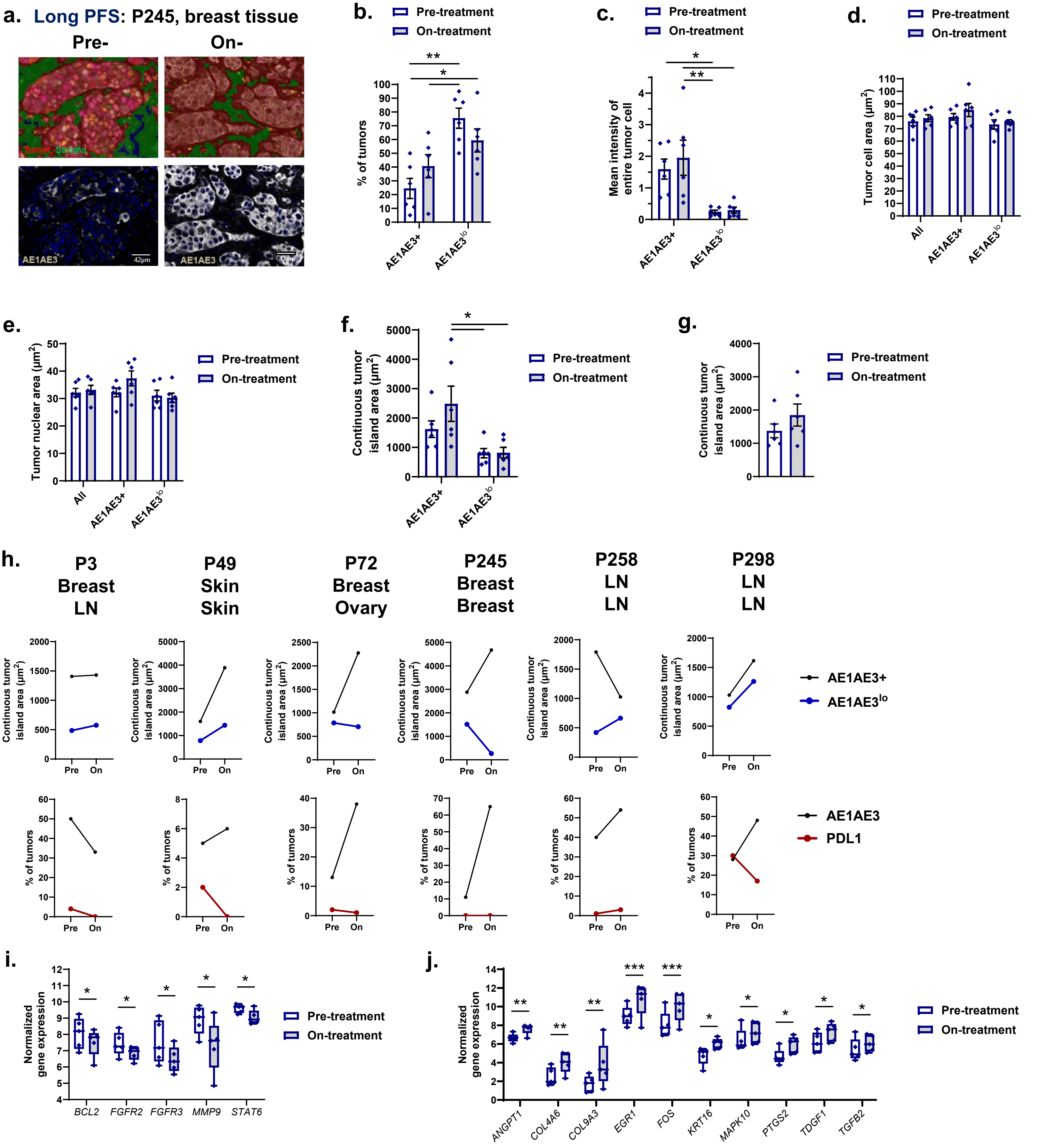

Supplementary Figure 4. Assessments of AE1/AE3 expression within treated tumors, Related to Figure 4. a Representative tissue segmentation and AE1/AE3 staining of pre- or on-treatment tissue from a patient with long PFS. Scale bar: 42µm. **b, c** Quantification of percentages (b) or mean intensities (c) of AE1/AE3 expression during treatment in comparison to pre-treatment. **d, e** Average tumor cell area (d) or nuclear area (e) of treated tissue compared to corresponding pre-treatment tissue. **f** Average AE1/AE3^{+} or AE1/AE3^{lo} tumor island area of corresponding pre- and on-treatment patient biopsies. **g** Overall assessment of continuous tumor island area during therapy. **h** Individual assessments of AE1/AE3 expression in relation to average continuous tumor island area (top) or tumor expression of PD-L1 (bottom) from matched pre- (listed first) and on-treatment tissue. **i, j** Select transcripts significantly lower among treated tissue (i) while growth factor or tissue structure gene expression increase during therapy (j). Data are represented as mean ± SEM. Significance was determined by Holm-Bonferroni correction for pre-planned multiple comparisons (b, c, f), unpaired t test with Welch's correction (d, e, g) or Wald test (i, j; n ≥ 5 patients/group). $*p<0.05$; $**p<0.01$; $***p<0.001$.

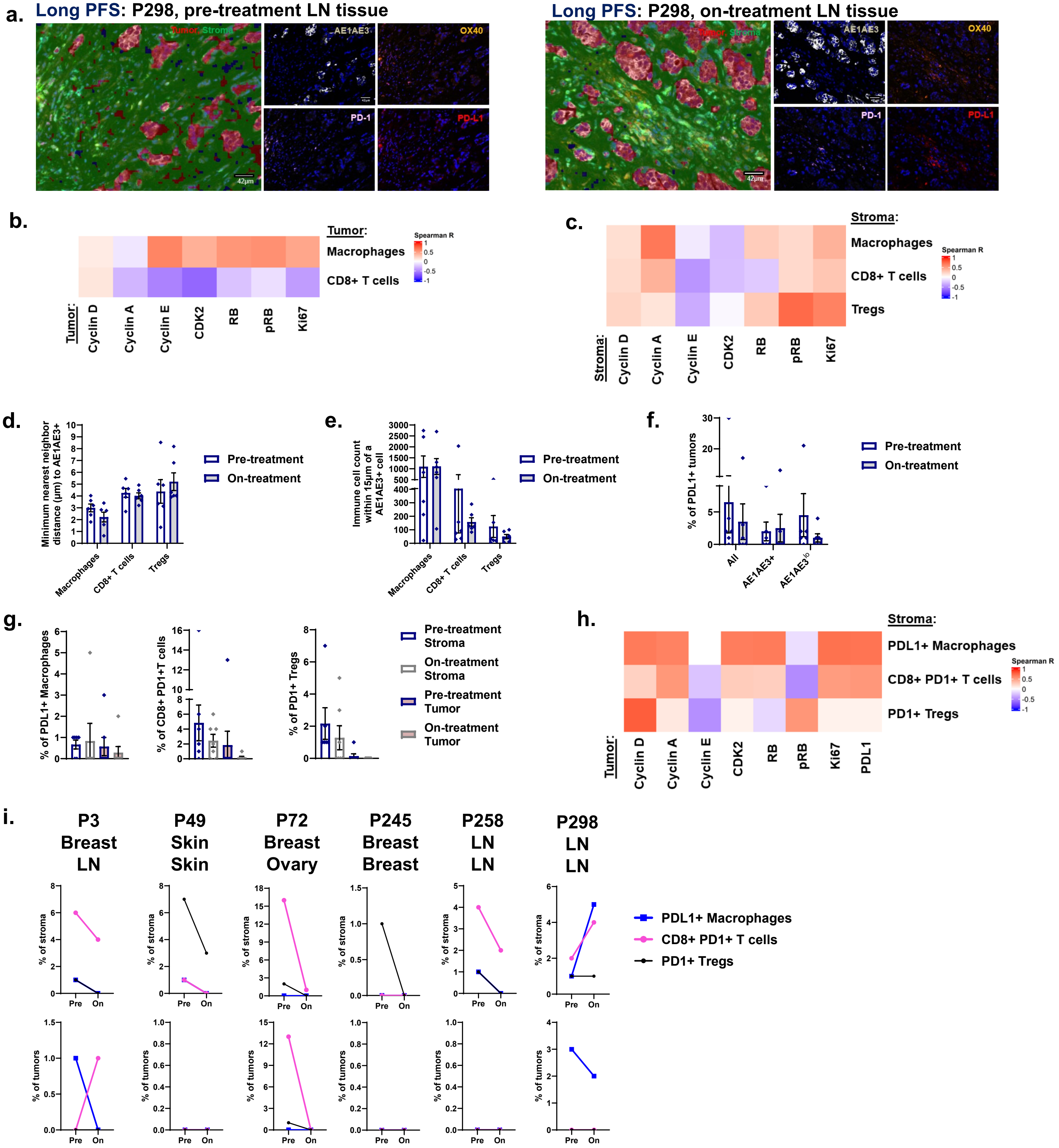

Supplementary Figure 5. Additional assessments of immune alteration during treatment, Related to Figure 5. a Representative AE1/AE3 and immune mIF staining of pre- or on-treatment tissue. Scale bar: 42µm. **b** Correlations of on-treatment tumor cell cycle proteins with intratumoral immune cells. **c** Correlations of on-treatment cell cycle proteins and immune cell detection within stroma. **d, e** Quantification of immune cell proximity to (d) and count within (e) a neighboring AE1/AE3^{+} tumor cell before and during treatment. **f, g** Percentages of tumors (f) and immune cells (g) with immune checkpoint molecule expression before and during treatment. **h** Correlations of on-treatment tumor cell cycle proteins with immune checkpoint molecule-expressing immune cells in stroma. **i** Individual assessments of stromal (top) or intratumoral (bottom) immune cells expressing immune checkpoint molecules from matched pre- and on-treatment tissue. Data are represented as mean ± SEM. Significance was determined by Spearman correlation (b, c and h), unpaired t test with Welch's correction (d-f) or Holm-Bonferroni correction for pre-planned multiple comparisons (g; n = 6 patients/group).

Supplementary Table 1: Characteristics of patients treated with CDK4/6i and with pre-treatment tissue analysis results. Treatment and disease status of metastatic HR+/HER2- breast cancer patients treated with CDK4/6i. *CDK4/6i* CDK4/6 inhibitor; *PFS* progression-free survival

**Patient Characteristics**	**N=155**
Months PFS (median)	11.90
CDK4/6i therapy (%)	
Palbociclib	144 (92.9)
Abemaciclib	2 (1.3)
Ribociclib	9 (5.8)
First-line	130 (83.9)
CDK4/6i status (%)	
Still on	32 (20.6)
Off due to progression	104 (67.2)
Off due to adverse effects	12 (7.7)
Off due to death	7 (4.5)
Endocrine therapy (%)	
Aromatase inhibitor	119 (76.8)
Fulvestrant	34 (21.9)
Tamoxifen	2 (1.3)
Race (%)	
African American	13 (8.4)
Caucasian American	135 (87.1)
Other	3 (1.9)
Not reported	4 (2.6)
Age at CDK4/6i start (mean)	61.14
Menopause status (%)	
Premenopausal	26 (16.8)
Menopausal	129 (83.2)
Metastatic status at presentation (%)	
De novo	56 (36.1)
Recurrent	99 (63.9)
Metastatic status (%)	
Non-visceral	77 (49.7)
Visceral	78 (50.3)
Prior chemotherapy (%)	76 (49.0)
Prior endocrine therapy (%)	90 (58.1)
Prior radiation therapy (%)	82 (52.9)

Supplementary Table 2: Summary of clinical sample number for transcriptomic or imaging analyses. Number of pre- or on-treatment biopsies utilized per analytical approach. *DSP* digital spatial profiling; *mIF* multispectral immunofluorescent; *RNA-seq* ribonucleic acid-sequencing

Analytical approach	Short PFS pre-treatment	Long PFS pre-treatment	Matched pre- & on-treatment
Targeted RNA-seq	17	63	5
mIF imaging	12	11	6
DSP imaging	-	-	7
